# Chromosome-level genome assembly of *Stellaria dichotoma* reveals extensive transposable element proliferation in a traditional Chinese medicinal plant

**DOI:** 10.3389/fpls.2026.1782751

**Published:** 2026-05-04

**Authors:** Xiao-Yan Gan, Sheng-Hu Guo, Xuan Liu, Yun-Zhe Dong, Hong-Jin Wang, Li Tian, Nan Zhong, Nadeem Bhanbhro, Li Peng, Yu-Chao Chen

**Affiliations:** 1 Agricultural Biotechnology Research Center, Ningxia Academy of Agriculture and Forestry Sciences, Yinchuan, Ningxia, China; 2 College of Life Sciences, Ningxia University, Yinchuan, Ningxia, China; 3 College of Life Sciences, Northwest A&F University, Shaanxi, Yangling, China; 4 Sanya Institute, Hainan Academy of Agricultural Sciences, Sanya, China

**Keywords:** genome assembly, LTR retrotransposons, *Stellaria dichotoma*, traditional Chinese medicine, whole-genome duplication

## Abstract

Stellaria Radix, known as Yinchaihu (YCH) in traditional Chinese medicine, is derived from the dried roots of *Stellaria dichotoma* L. var. *lanceolata* Bge. Despite its clinical importance in treating pediatric febrile conditions and documented pharmacological activities, comprehensive genomic resources for this species have been unavailable. Here, we report the first chromosome-level genome assembly of YCH generated using PacBio HiFi long-read sequencing integrated with Hi-C chromatin conformation capture technology. The assembled genome spans 1.93 Gb with a contig N50 of 125.08 Mb and scaffold N50 of 133.88 Mb. Hi-C-assisted scaffolding anchored 1.93 Gb (99.98%) of assembled sequences onto 14 chromosomes, achieving 96.53% BUSCO completeness, a Long Terminal Repeat Assembly Index (LAI) of 17.04, and a quality value (QV) of 29.47, confirming high assembly quality. Genome annotation identified 48,391 protein-coding genes, with 95.51% receiving functional annotation across multiple databases. Repetitive sequences constitute 84.46% of the genome, predominantly LTR retrotransposons (67.44%), with Gypsy (39.13%) and Copia (14.18%) elements dominating. Ks distribution analysis revealed a shared ancient whole-genome duplication (WGD) event across Caryophyllaceae species, with peak LTR retrotransposon insertion activity at approximately 0.5 million years ago coinciding temporally with the WGD, suggesting polyploidy-triggered transposon proliferation. This high-quality reference genome provides essential genetic resources for elucidating the molecular mechanisms underlying the medicinal properties of YCH and will facilitate future molecular breeding, functional genomics, and pharmacological investigations of this important medicinal plant.

## Introduction

**Figure 1:**
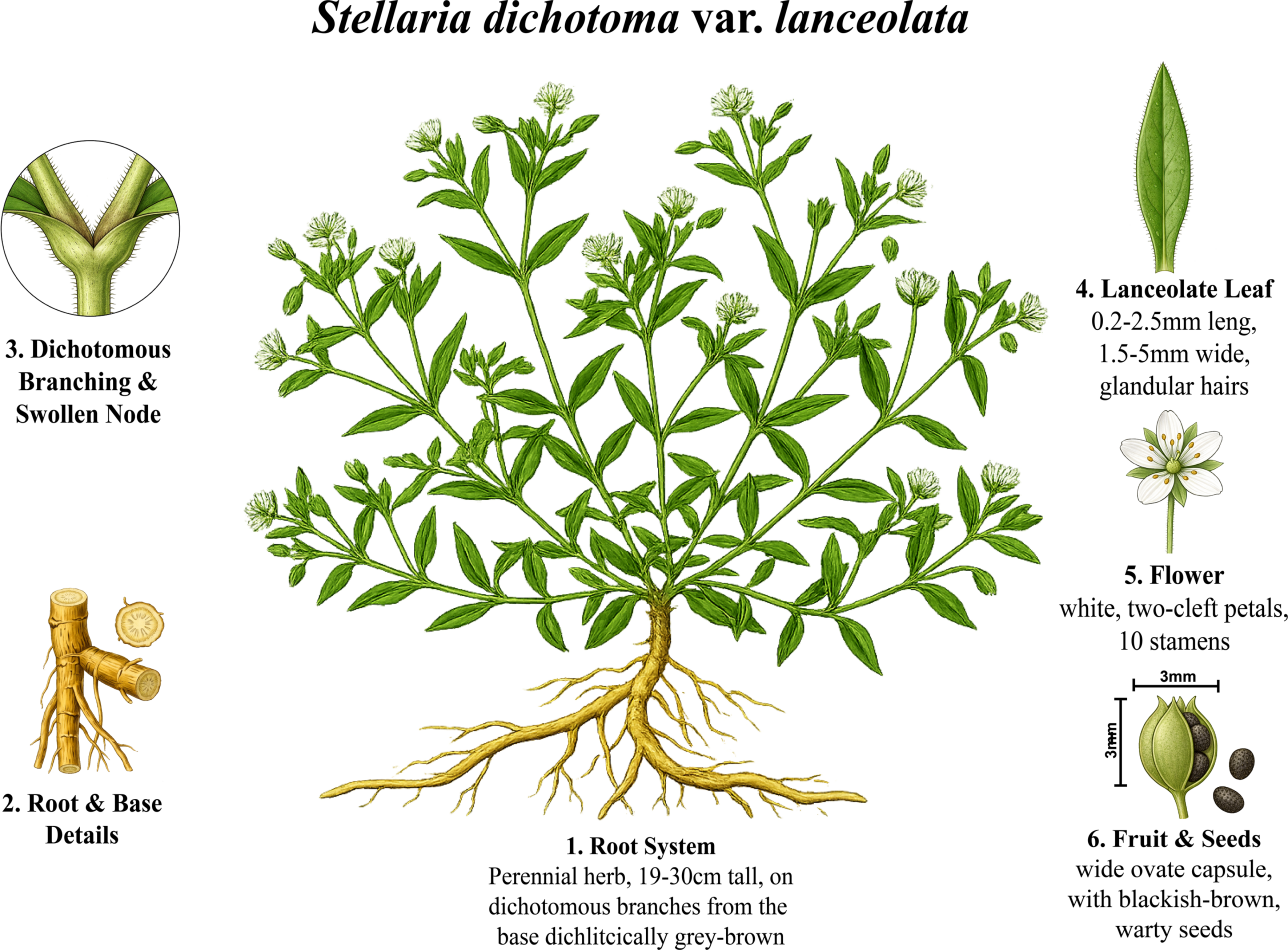
Morphological illustration of *Stellaria dichotoma* var. lanceolata showing: (1) overall habit and root system; (2) root and stem base details; (3) dichotomous branching with swollen nodes; (4) lanceolate leaf with glandular hairs; (5) white flower with two-cleft petals and 10 stamens; and (6) wide ovate fruit capsule with blackish-brown warty seeds.

Yinchaihu (Stellaria Radix, 2n=2x=28) is a perennial herb belonging to the Caryophyllaceae family, traditionally employed in Chinese medicine for treating deficiency-related fever (Bae et al., 2018; Li et al., 2020). As a typical xerophytic species, YCH naturally inhabits rocky slopes at elevations of 1,200–3,000 m and thrives in infertile sandy soils (Zhao et al., 2020). *Stellaria dichotoma* var. lanceolata is a perennial herb (19–30 cm tall) exhibiting distinctive morphological features, including a dichotomously branched spreading habit with swollen nodes, a robust taproot system, lanceolate leaves (0.2–2.5 mm long, 1.5–5 mm wide) bearing glandular hairs, white flowers with two-cleft petals and 10 stamens, wide ovate capsules (~3 × 3 mm), and blackish-brown warty seeds (**Figure 1**). The flowering period extends from May to June, with fruit maturation occurring from July to August (Zhao et al., 2020). Clinically, YCH treats pediatric high fever and demonstrates efficacy against allergic diseases, cancer, and vascular disorders (Tan and Zhou, 2006; Jia et al., 2007). Its bioactive constituents include sterols (Morikawa et al., 2004), alkaloids (Sun et al., 2004; Luo et al., 2012), and flavonoids (Li et al., 2023). Notably, α-spinasterol, abundant in YCH, exhibits anti-inflammatory and antipyretic properties and represents one of the main bioactive components (Li et al., 2022). The 70% ethanol extract reduces mortality in Mycobacterium abscessus-infected mice while mitigating associated inflammatory responses (Bae et al., 2018). Multiple alkaloids within YCH possess potent anti-inflammatory activity (Cui et al., 2017). Although clinical and pharmacological studies confirm YCH antipyretic efficacy, the molecular basis and mechanisms of action remain incompletely characterized. Several genome assemblies have been reported for species within the Caryophyllaceae family, including *Dianthus caryophyllus* (carnation) (Jiang et al., 2023), *Gypsophila paniculata* (baby’s breath) (Li et al., 2022), and *Silene latifolia* (white campion) (Moraga et al., 2025). However, no genome assembly has been reported for the genus Stellaria to date, representing a significant gap in genomic resources for this medicinally and ecologically important group.

To address this knowledge gap and provide the first genomic resource for the genus *Stellaria*, we performed *de novo* chromosome-level genome assembly and annotation of YCH using PacBio HiFi sequencing and Hi-C technology, establishing a foundation for investigating the molecular mechanisms underlying its key medicinal components.

## Materials and methods

### Plant materials and sequencing

Fresh leaves of *Stellaria dichotoma* were collected from Lingwu in Ningxia Hui Autonomous Region, China. The fresh young leaves were placed in a cryogenic vial with liquid nitrogen and stored at −80 °C. High-molecular-weight genomic DNA (gDNA) was extracted from young leaves using the cetyltrimethylammonium bromide (CTAB) method (Winnepenninckx et al., 1993). The quality and quantity of the extracted DNA were evaluated using agarose gel electrophoresis and a spectrophotometer.

PacBio HiFi sequencing was performed using the SMRTbell Express Template Prep Kit 2.0. The library construction process included DNA fragmentation, end repair, damage repair, and adapter ligation. The purified libraries were sequenced on the PacBio platform, generating a total of 153.48 Gb of HiFi clean data. Based on the estimated genome size of 1.85 Gb, this corresponds to approximately 83 × coverage. The average length of HiFi reads reached 17.46 Kb, providing excellent continuity for genome assembly (**Table 1**).

**Table 1:** Summary of raw and clean Hi-C sequencing data quality metrics.

Sample	Hi-C-01	Hi-C-02
Raw Reads Number	1,145,781,244	1,275,594,092
Raw Bases Number	343,291,570,348	191,339,113,800
Clean Reads Number	891,427,252	1,140,753,160
Clean Reads Rate (%)	85.27	89.43
Clean Bases Number	133,714,087,800	171,112,974,000
Raw Q30 Bases Rate (%)	93.86	95.64
Clean Q30 Bases Rate (%)	95.41	96.21
Raw GC percent (%)	38.9	40.36
Clean GC percent (%)	40.07	40.05

For Hi-C library construction, DNA was cross-linked to preserve spatial interaction relationships and three-dimensional chromatin structure. DNA was digested with HindIII restriction enzyme, and fragments of 300–700 bp were recovered. Interacting DNA fragments were captured using streptavidin immunomagnetic beads. Library quality was assessed using Qubit 3.0 and GX platform, and quantitative PCR (qPCR) was used to quantify library concentration. Hi-C sequencing was performed on the Illumina platform, generating approximately 305 Gb of clean data (**Supplementary Table 1**).

A small-fragment sequencing library was constructed with a target fragment size of 350 bp, followed by end-repair, A-tailing, adapter ligation, and PCR. The library was sequenced on the Illumina platform, generating paired-end 150 bp (PE150) reads. Sequencing data were evaluated and filtered to obtain high-quality clean reads using FastQC and Trimmomatic (Bolger et al., 2014). These clean reads were used for genome-size estimation, genome assembly, GC-content analysis, and heterozygosity rate calculation.

### Genome assembly and quality assessment

*De novo* genome assembly was performed using Hifiasm v0.19 (Cheng et al., 2021) with default parameters. Hi-C-assisted pseudochromosome construction was performed using LACHESIS (Servant et al., 2015) for scaffolding, ordering, and orientation, followed by manual heatmap adjustment. HiC-Pro v2.10.0 (Servant et al., 2015) was used to process Hi-C data, and BWA v0.7.17 (Li and Durbin, 2009) was used to align sequencing reads to the contig sequences. Genome completeness was assessed using BUSCO v5.2.1 (Manni et al., 2021) against the embryophyta_odb10 database. Assembly quality value (QV) was estimated using Merqury v1.3 (Rhie et al., 2020). Briefly, k-mers (k = 21) were extracted from the HiFi reads using Meryl (https://github.com/marbl/meryl) and used to build a k-mer database. The k-mer spectrum was then compared between the reads and the assembly to identify k-mers present exclusively in the reads (read-only k-mers), which represent sequencing errors. QV was calculated as QV = −10 × log10(error rate), where the error rate was estimated as the proportion of read-only k-mers relative to total k-mers in the reads. The Long Terminal Repeat Assembly Index (LAI) was calculated using LTR_retriever v2.9.0 (Ou and Jiang, 2018a) to evaluate assembly continuity and reliability. Illumina reads were mapped to the assembly using BWA (Li and Durbin, 2009) to assess read mapping rates.

### Repeat annotation

*De novo* repeat prediction was performed using RepeatModeler2 v2.0.1 (Chen, 2004), incorporating RECON v1.0.8 (Beier et al., 2017) and RepeatScout v1.0.6 (Benson, 1999), with RepeatClassifier and Dfam v3.5 for classification. LTR_retriever v2.9.0 (Ou and Jiang, 2018a) identified LTR retrotransposons through LTRharvest v1.5.10 (Stanke et al., 2008) and LTR_FINDER v1.0.7 (Korf, 2004). A species-specific repeat library was constructed by integrating *de novo* predictions with known databases after redundancy removal. RepeatMasker v4.1.2 (Keilwagen et al., 2016), was used to identify transposable elements (TEs) using this custom library. Tandem repeats were predicted using MISA v2.1 (Grabherr et al., 2011) and Tandem Repeat Finder (TRF v409) (Haas et al., 2003).

### Gene prediction and functional annotation

Coding gene prediction was performed using three complementary approaches: homology-based, *de novo*, and transcriptome-based prediction.

*De novo* prediction was performed using Augustus v3.1.0 (Haas et al., 2008), and SNAP (Lowe and Eddy, 1997). Homology-based prediction was conducted using GeMoMa v1.7 (Loman, 2017) with protein sequences from four Caryophyllaceae species (*Dianthus caryophyllus, Gypsophila paniculata, Silene conica, Gypsophila przewalskii*), *Sorghum bicolor*, and *Arabidopsis thaliana*. Transcriptome-based prediction was performed using RNA-seq data assembled via Hisat v2.1.0 (Griffiths-Jones et al., 2005), and Stringtie v2.1.4 (Nawrocki and Eddy, 2013), and predicted with GeneMarkS-T v5.1 (She et al., 2009), or assembled with Trinity v2.11 (Birney et al., 2004), and predicted using PASA v2.4.1 (Huerta-Cepas et al., 2019). PacBio Iso-Seq data were aligned using GMAP and processed through PASA. Evidence Modeler v1.1.1 (Kanehisa et al., 2016), integrated all predictions, with PASA performing final refinement.

Functional annotation of protein-coding genes was performed using NR, eggNOG (Boeckmann et al., 2003), GO, KEGG (Finn et al., 2006), TrEMBL (Finn et al., 2006), KOG, SWISS-PROT (Emms and Kelly, 2019) and Pfam (Mi et al., 2019) databases.

### Whole-genome duplication analysis

To investigate whole-genome duplication (WGD) events, synonymous substitution rates (Ks) were calculated for homologous gene pairs within and between *S. dichotoma* and related Caryophyllaceae species: *S. latifolia, S. conica, Gypsophila przewalskii, G. paniculata*, and *Dianthus caryophyllus*. Protein sequences were aligned using BLAST and processed with MCScanX. Ks values were calculated using the Nei-Gojobori method implemented in KaKs_Calculator v2.0. Ks distribution plots were generated using R.

### LTR retrotransposon insertion age analysis

Full-length long terminal repeat retrotransposons (fl-LTR-RTs) were identified using both LTRharvest v1.5.10 (Stanke et al., 2008) and LTR_FINDER v1.0.7 (Korf, 2004). High-quality intact fl-LTR-RTs and a non-redundant LTR library were produced using LTR_retriever v2.9.0 (Ou and Jiang, 2018a). The flanking sequences on both sides of LTRs were extracted, aligned using MAFFT v7.205 (Nguyen et al., 2015) and the divergence distance was calculated using the Kimura model in EMBOSS v6.6.0 (Yang, 1997). LTR retrotransposon insertion ages were estimated using the formula: T = K/2μ, where K is the divergence rate between LTR sequences and μ is the substitution rate (assumed to be 7.0 × 10^-9^ substitutions per site per year).

## Results

### Genome survey and size estimation

**Figure 2:**
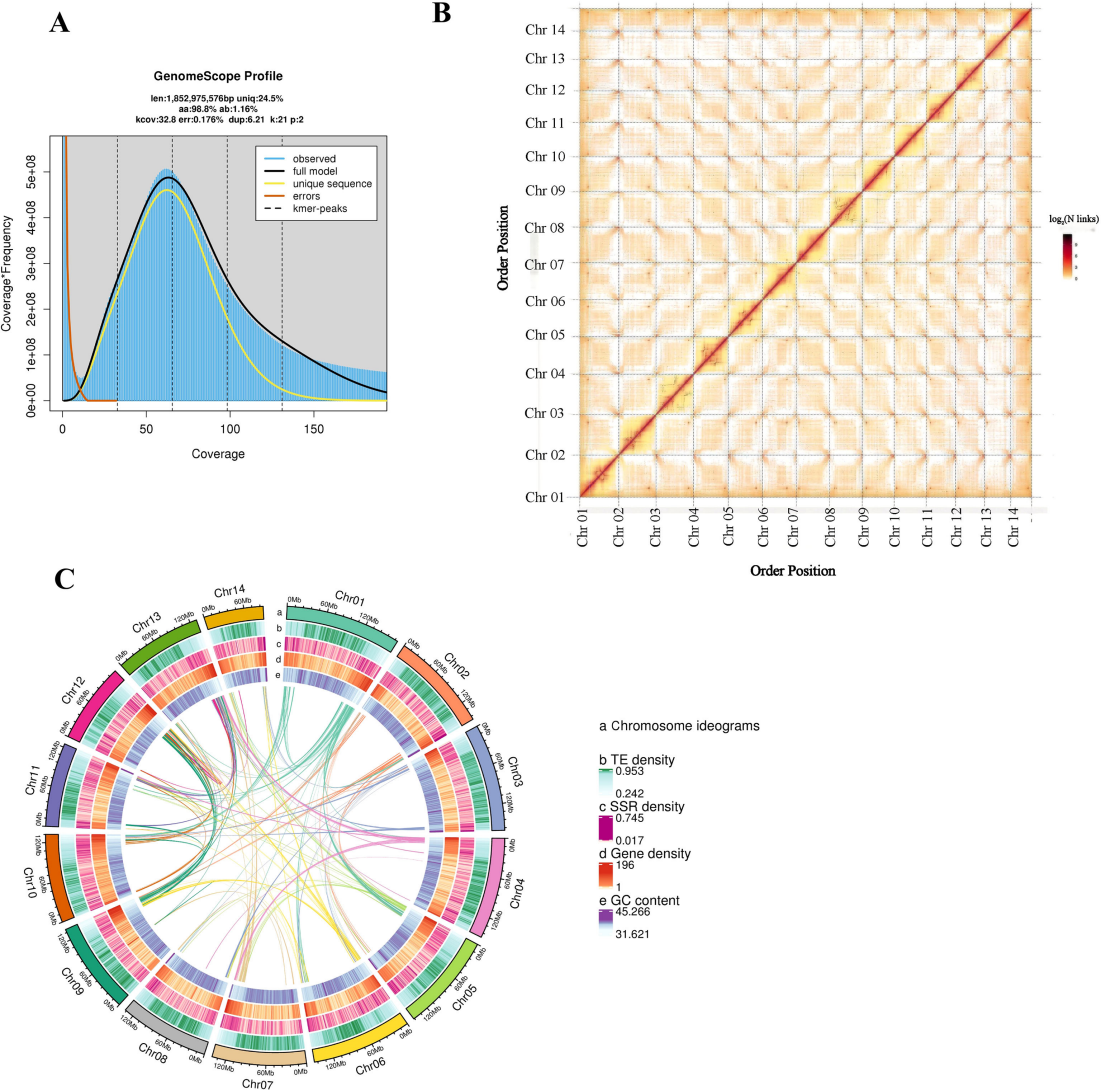
Genomic characteristics of *S. dichotoma*. **(A)** K-mer frequency distribution curve. **(B)** Hi-C interaction heatmap showing chromatin contacts across the 14 chromosomes (denoted by red boxes along the diagonal). **(C)** Circos plot displaying genomic features including chromosome ideograms, transposable element (TE) density, simple sequence repeat (SSR) density, gene density, GC content, and syntenic relationships among chromosomes.

We generated 343.29 Gb of Illumina clean data for genome characterization (**Table 1**). K-mer frequency distribution analysis using GenomeScope estimated genome size at 1.85 Gb, heterozygosity at 1.16%, and repetitive content at 84.46% (**Figure 2A**; **Table 1**), classifying this as a highly heterozygous complex genome.

### *De novo* genome assembly

HiFi data were assembled using Hifiasm v0.19 (Cheng et al., 2021), yielding a 1.93 Gb assembly with scaffold N50 of 125.08 Mb (**Tables 1**, **2**). BUSCO v5.2.1 (Manni et al., 2021) assessment against the OrthoDB10 embryophyta database demonstrated 96.53% completeness (**Tables 2**, **3**), confirming high genome integrity. Assembly quality value (QV) was estimated using Merqury v1.3 (Rhie et al., 2020) based on k-mer analysis of the HiFi reads, yielding a QV of 29.47. For pseudochromosome construction, Hi-C-assisted assembly employed LACHESIS software for scaffolding, ordering, and orientation, followed by manual heatmap adjustment. This anchored 1.93 Gb (99.98%) onto 14 chromosomes (**Figures 2B, C**; **Table 4**). The Long Terminal Repeat Assembly Index (LAI) of 17.04 exceeded the reference genome quality threshold (10 ≤ LAI < 20) (Ou et al., 2018), validating assembly continuity and reliability.

**Table 2:** Summary of the YCH genome assembly and annotation.

Genome assembly	Value
Total length of assembly (Gb)	1.93
Number of contigs	275
Contig N50 (Mb)	125.08
Pseudochromosome number	14
Scafold N50 (Mb)	133.88
Number of gaps	25
Anchor rate (%)	99.98
Mapped Illumina reads (%)	99.17
BUSCO (%)	96.53
GC content (%)	37.54
LAI	17
QV	29.47
Genome annotation	
Total length of repeats (Mb)	1560.15
Repeats percentage of assembly (%)	84.45
Number of protein-coding genes	48,391
Average gene length (bp)	580.26
Average intron length (bp)	2669.23
Average exon length (bp)	1911.03
Functional annotation	
SwissProt	30,803(63.65%)
Nr	44,465(91.89%)
GO	35,945(74.28%)
Pfam	39,224(81.06%)
eggNOG	39,136(80.87%)
KEGG	36,108(74.62%)
KOG	28,482(58.86%)
TrEMBL	45,665(94.37%)
Total	46,216(95.51%)

**Table 3:** BUSCO assessment of *Stellaria dichotoma* genome assembly.

Item	Assembly (embryophyte)	
	Number	Percent (%)
Complete BUSCOs (C)	1544	0.9566
Complete and single-copy BUSCOs (S)	1478	0.9157
Complete and duplicated BUSCOs (D)	66	0.0409
Fragmented BUSCOs (F)	15	0.0093
Missing BUSCOs (M)	55	0.0341
Total BUSCO groups searched	1614	1

**Table 4:** Chromosome assembly statistics.

Chromosome	Cluster num	Length (bp)	Contig
Chr01	11	166,920,049	2
Chr02	11	160,360,840	2
Chr03	14	160,065,921	4
Chr04	8	148,943,283	3
Chr05	16	146,682,989	4
Chr06	4	145,136,399	2
Chr07	6	141,213,088	3
Chr08	11	140,423,848	4
Chr09	4	137,708,571	2
Chr10	10	137,622,680	2
Chr11	4	124,598,378	1
Chr12	7	124,571,865	1
Chr13	3	113,073,782	2
Chr14	4	89,461,262	2
Total	113	1,936,782,955	34

### Repeat annotation and transposable element landscape

Repeat annotation revealed that 84.46% (1.64 Gb) of the *S. dichotoma* genome comprises repetitive sequences, including 80.52% (1.56 Gb) transposable elements (**Figure 2B**; **Table 5**). LTR retrotransposons dominated the TE landscape at 67.44%, with Gypsy (758.04 Mb, 39.13%) and Copia (274.61 Mb, 14.18%) superfamilies being most abundant. Non-LTR retrotransposons constituted 0.84%, while Class II DNA transposons represented 12.23%, including Helitron elements (155.56 Mb, 8.03%). Tandem repeats accounted for 3.94% (76.27 Mb) of the genome (**Table 5**). A total of 1,482,624 TE copies were annotated across the genome (**Table 6**). Class I retroelements comprised 999,960 copies (1.32 Gb, 68.28%), with Gypsy representing the largest component at 461,368 copies (758.04 Mb, 39.13%). Copia elements accounted for 164,980 copies (274.61 Mb, 14.18%), while LTR/Unknown categories contributed 321,461 copies (267.28 Mb, 13.80%). Non-LTR retrotransposons were sparse, with LINEs comprising 41,451 copies (15.51 Mb, 0.80%) and SINEs 4,848 copies (0.70 Mb, 0.04%). Class II DNA transposons totaled 482,647 copies (236.98 Mb, 12.23%). Helitron elements dominated this class with 322,970 copies (155.56 Mb, 8.03%). Other families, including CACTA (13,783 copies, 1.12%), Mutator (4,243 copies, 0.16%), hAT (3,306 copies, 0.12%), and PIF-Harbinger (1,114 copies, 0.02%), contributed minimally (**Table 6**).

**Table 5:** Repeat content of the YCH genome.

Class	Length (bp)	Percentage in genome (%)
Class I: Retrotransposon	1,308,100,670	68.28
LTR Retrotransposon	1,306,479,527	67.44
Copia	274,611,886	14.18
Gypsy	758,044,056	39.13
Others	273,823,585	14.13
LTR Retrotransposon	16,211,432	0.84
LINE	15,512,658	0.8
**Class II: DNA Transposon**	236,975,727	12.23
PIF-Harbinger	155,557,669	0.02
Maverick	3,126,408	0.01
Mutator	2,358,109	0.16
PIF-Harbinger	479,345	0.02
TcMar-Pogo	97,270	0
hAT-Ac	5,953	0.12
Helitron	1,559,667,822	8.03
Total TE	76,267,978	80.52
Tandem repeat	1,635,935,800	3.94
Total repetitive sequences	1,308,100,670	84.46

Bold text indicates the Class II: DNA transposon category

**Table 6:** Detailed transposable element annotation.

Type	Number	Length	Rate (%)
**Class I: Retroelements**	999960	1322690959	68.28
LINE	41451	15512658	0.8
LTR/Caulimovirus	3952	6289654	0.32
LTR/Copia	164980	274611886	14.18
LTR/ERV	1296	133386	0.01
LTR/Gypsy	461368	758044056	39.13
LTR/Ngaro	299	41471	0
LTR/Pao	305	76257	0
LTR/Unknown	321461	267282817	13.8
SINE	4848	698774	0.04
**Class II: DNA Transposons**	482647	236975727	12.23
Academ	2	74	0
CACTA	13783	21767532	1.12
Crypton	17	822	0
Dada	104	5053	0
Ginger	77	5446	0
Helitron	322970	155557669	8.03
IS3EU	214	38234	0
Kolobok	510	56595	0
Maverick	287	97270	0.01
Merlin	104	3864	0
Mutator	4243	3126408	0.16
P	57	3941	0
PIF-Harbinger	1114	479345	0.02
PiggyBac	60	2318	0
Tc1-Mariner	115	5953	0
Unknown	135676	53466728	2.76
Zisupton	8	366	0
hAT	3306	2358109	0.12
Unknown	17	1136	0
Total	1482624	1559667822	80.52

Bold text indicates the Class II: DNA transposon category

### Gene prediction and functional annotation

Gene prediction integrated three complementary approaches: homology-based prediction using (Keilwagen, et al., 2016; Bae et al., 2018), GeMoMa v1.7, with protein sequences from four Caryophyllaceae species (*Dianthus caryophyllus*, *Gypsophila paniculata*, *Silene conica*, *Gypsophila przewalskii*), *Sorghum bicolor*, and *Arabidopsis thaliana*; *de novo* prediction using Augustus v3.1.0 (Stanke et al., 2008), SNAP (Korf, 2004) and transcriptome-based prediction from RNA-seq data assembled via Hisat v2.1.0 (Grabherr et al., 2011), and Stringtie v2.1.4 (Pertea et al., 2015) and predicted with GeneMarkS-T v5.1 (Tang et al., 2015), or assembled with Trinity v2.11 (Grabherr et al., 2011) and predicted using PASA v2.4.1 (Xu and Wang, 2007). PacBio Iso-Seq data were aligned using GMAP and processed through PASA. EVidence Modeler v1.1.1 (Haas et al., 2008), integrated all predictions, with PASA performing final refinement, yielding 48,391 protein-coding genes with average gene length of 2,669.23 bp, exon length of 580.26 bp, and intron length of 1,911.03 bp (**Figure 2C**; **Table 2**). Functional annotation achieved 95.51% coverage across NR (91.89%), GO (74.28%), KEGG (74.62%), Swiss-Prot (63.65%), Pfam (81.06%), eggNOG (80.87%), KOG (58.86%), and TrEMBL (94.37%) databases (**Table 2**; **Supplementary Table 2**).

### Whole-genome duplication and LTR dynamics

**Figure 3:**
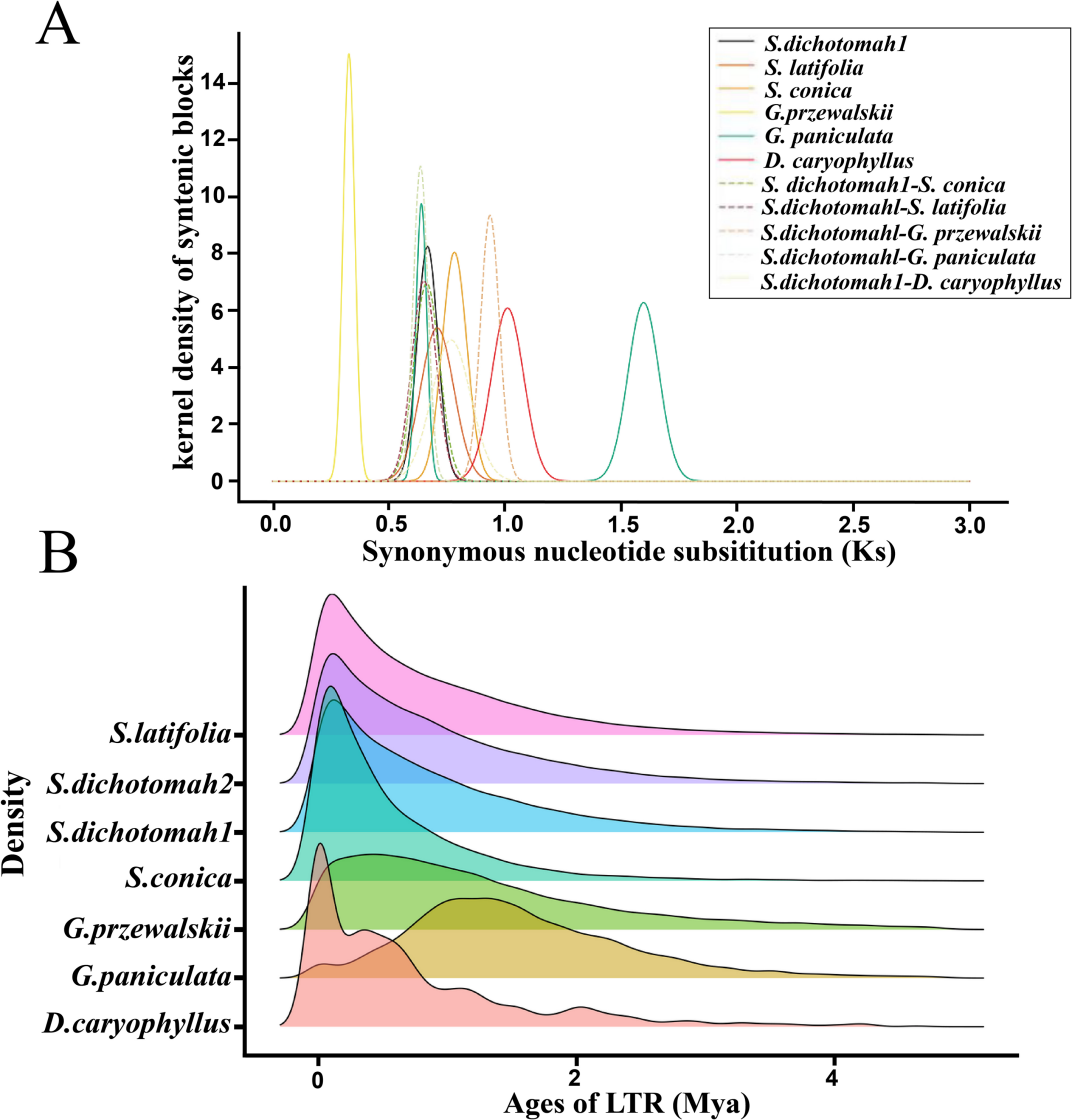
Genome evolution of *Stellaria dichotoma*. **(A)** Ks distribution plots showing synonymous substitution rates for paralogous and orthologous gene pairs in *S. dichotoma, S. latifolia, S. conica, G. przewalskii, G. paniculata*, and *D. caryophyllus*. The peak at Ks = 0.66 in *S. dichotoma* and concordant peaks in related species indicate a shared whole-genome duplication (WGD) event. **(B)** Insertion ages of LTR retrotransposons in *S. dichotoma, S. latifolia, S. conica, G. przewalskii, G. paniculata*, and *D. caryophyllus*. Mya: million years ago.

To investigate whole-genome duplication (WGD) events in *Stellaria dichotoma*, we calculated synonymous substitution rates (Ks) for homologous gene pairs within and between six Caryophyllaceae species: *S. dichotoma, S. latifolia, S. conica, Gypsophila przewalskii, G. paniculata*, and *Dianthus caryophyllus*. Ks distribution analysis revealed a prominent peak at 0.66 in *S. dichotoma*, while orthologous gene pairs between *S. dichotoma* and G. paniculata showed a significant peak at Ks = 0.63. Additionally, concordant Ks peaks were observed across other Caryophyllaceae species within a range of 0.65–0.93 (**Figure 3A**), indicating a shared WGD event in the family’s evolutionary history.

LTR retrotransposon content varied among species, with *S. dichotoma* exhibiting the highest proportion (LTRs: 84.34%; Gypsy: 54.05%), compared to S. conica (LTRs: 81.90%; Gypsy: 34.01%) and *S. latifolia* (LTRs: 67.67%; Gypsy: 42.04%) (**Supplementary Table 3**). Analysis of LTR insertion ages revealed that most insertions occurred within the past 0–2.0 million years (Mya) (**Figure 3B**). Peak LTR activity was observed at approximately 0.5 Mya in *S. dichotoma, S. latifolia, and S. conica*. In contrast, *G. przewalskii* showed peak activity at 1.5 Mya, while G. paniculata exhibited a later peak at 2.0 Mya (**Figure 3B**). The temporal overlap between the shared WGD event (Ks peak at ~0.66) and peak LTR activity (~0.5 Mya) in *S. dichotoma* suggests polyploidy-triggered transposon activation.

## Discussion

### High-quality genome assembly reveals complex genomic architecture

The first chromosome-level assembly of *S. dichotoma* spans 1.85 Gb (contig N50: 125.08 Mb; scaffold N50: 133.88 Mb) with 99.98% of sequences anchored onto 14 chromosomes. Despite high heterozygosity (1.16%) and repetitive content (84.46%), integration of PacBio HiFi and Hi-C data successfully resolved complex regions, achieving 96.53% BUSCO completeness (Manni et al., 2021), an LAI of 17.04, and a QV of 29.47 (Rhie et al., 2020). These metrics surpass all currently available Caryophyllaceae genomic resources and demonstrate the utility of long-read sequencing for highly repetitive plant genomes.

### LTR retrotransposon proliferation drives genome expansion

The large genome size reflects extensive transposable element amplification, particularly LTR retrotransposons (67.44%). Gypsy elements dominate (758.04 Mb, 39.13%), exceeding Copia (274.61 Mb, 14.18%) by 2.76-fold. Comparative analysis reveals lineage-specific *dynamics: S. dichotoma* harbors >35,000 LTRs per haplotype, substantially more than *Dianthus caryophyllus* (963) or *Gypsophila paniculata* (7,409) but fewer than *Silene latifolia* (58,767) (**Table 7**). Insertion time analysis indicates most activity occurred within the past 2.0 million years, peaking at ~0.5 million years ago, consistent with recent transposon bursts observed in other plant genomes with large, repeat-rich architectures, contributing to genomic plasticity and adaptive evolution.

**Table 7:** Number of LTR retrotransposon elements identified across Caryophyllaceae species.

Category	*G. przewalskii*	*S. dichotoma* H1	*S. dichotoma* H2	*S. conica*	*S. latifolia*	*G. paniculata*	*D. caryophyllus*
Copia	25398	10747	10841	6955	15061	1799	525
Gypsy	5453	19178	19123	4939	24706	4499	190
unknown	2945	5557	5392	2629	19000	1111	248
Total	33796	35482	35356	14523	58767	7409	963

### Ancient whole-genome duplication shapes genomic architecture

The shared Ks peak at 0.66 across Caryophyllaceae species provides strong evidence for a common whole-genome duplication (WGD) event in the evolutionary history of this family (**Figure 3A**). The temporal overlap between this WGD event and peak LTR retrotransposon activity at approximately 0.5 million years ago (**Figure 3B**) suggests polyploidy-triggered transposon activation (Haas et al., 2003), a well-documented mechanism for genomic restructuring and functional innovation. Such WGD-LTR coupling has been widely reported in flowering plants and likely contributed to the expansion of gene families involved in specialized metabolite biosynthesis, potentially shaping the medicinal properties of *S. dichotoma.*

### Adaptive gene family evolution in metabolic pathways

Comparative analysis identified 829/797 expanded and 487/516 contracted gene families across haplotypes. Expansions significantly enrich photosynthesis, carbon fixation, and secondary metabolism pathways, particularly unsaturated fatty acid biosynthesis and terpenoid-quinone metabolism, pathways directly linked to anti-inflammatory and antipyretic compound production (Price et al., 2005; Cheng et al., 2021). The 2,353/2,237 species-specific genes (448/437 orthogroups) (**Supplementary Table 4**) show enrichment in heterocycle biosynthesis and nucleobase-containing compound metabolism, representing promising candidates for novel medicinal compounds (Li et al., 2020, 2023).

### Molecular authentication and quality control applications

The chromosome-level assembly and species-specific orthogroups provide robust resources for developing PCR-based authentication markers. These tools enable discrimination of authentic *S. dichotoma* from common adulterants (*Stellaria media*), addressing critical quality control needs in traditional Chinese medicine supply chains (Morikawa et al., 2004; Luo et al., 2012; Li et al., 2020), and ensuring therapeutic consistency.

## Conclusion

This study presents the first chromosome-level genome assembly of *Stellaria dichotoma*, spanning 1.85 Gb with 99.98% of sequences anchored across 14 chromosomes, achieving 96.53% BUSCO completeness, an LAI of 17.04, and a QV of 29.47. The genome provides critical resources for understanding the genetic architecture and molecular mechanisms underlying its medicinal properties, facilitating research on bioactive compound biosynthesis and crop improvement. The genome reveals a history of whole-genome duplication and recent transposon proliferation, with LTR retrotransposons accounting for 67.44% of the 1.85 Gb genome and insertion activity peaking at ~0.5 Mya, that shaped its large, complex structure, while gene family expansions in metabolic pathways provide a genomic foundation for therapeutic efficacy. Together, these findings establish *S. dichotoma* as a genomic model for the genus *Stellaria* and will accelerate molecular breeding, authentication, and pharmacological investigation of this important medicinal plant.

## Data Availability

These data have been deposited in the Genome Sequence Archive In National Genomics Data Center, China National Center for Bloinformation/Beljing Institute of Genomics, Chinese Academy of Sciences (GSA : CRX1299052, CRX1299053, CRX1299054, CRX1299056 and CRX1299056) that are publicly accessible at https://ngdc.cncb.ac.cn/gsa. The genotype data are available in NGDC under project number PRJCA033494.
